# Abstract of the Proceedings of the Buffalo Medical Association

**Published:** 1864-11

**Authors:** Joseph A. Peters

**Affiliations:** Secretary


ART. II—*Abstract of the Proceedings of the Buffalo Medical Association.*

TUESDAY EVENING, October 4th, 1864.

Society met pursuant to adjournment, the President, Dr. Samo, being in the chair. Present, Drs. Rochester, Lockwood, Miner, Strong, Shaw, Congar, Boardman, Cronyn, Gay, Wyckoff, Johnson and Peters.

Minutes of last meeting read and approved.

*Dr. Hauenstein* was elected a member.

*Dr. Rochester* wished to call attention to the prevalence of a low fever, which might be called typhus or typhoid, but which was certainly not the typhoid fever of Louis. It did not present any enlargement of Peyer's glands, nor, usually, any diarrhœa or rose-colored eruption. It attacked children younger than were generally liable to typhoid fever. It commenced much like remittent fever, with pains in the back, limbs anorexia, &c., yet it could not be called simple remittent. The tongue was glabrous, sometimes cracked. There was often delirium, as in remittent; remissions there certainly were, but of a low type. These cases do not usually bear quinine well at first, but after the irritated condition of the stomach was subdued, quinine came in very nicely. It seemed to be, in short, a gastric form of remittent, of a low type. He had also noticed in connection with this and dysentery, an inflammation of the parotid and submaxiliary glands.

*Dr. Miner* had almost come to doubt the constancy of diarrhœa or rose-colored eruptions as symptoms of typhoid fever; they do not accompany a majority of cases in districts where there is no malaria and pure typhoid fever is prevalent; on the contrary, laxatives have often to be given. Diarrhœa and eruptions are often present, but their absence forms no doubt as to the character of the disease.

*Dr. Peters* had seen, in camp, many cases of a fever which presented precisely the same symptoms as Dr. Rochester had described, and which was called by some surgeons typhoid, but is now generally known in the army reports, as typho-malarial fever. Had noticed all the peculiarities described by Dr. Rochester, and had also had occasion to notice the extreme rapidity with which convalescence was established after the fever was somewhat subdued.

*Dr. Rochester* had noticed the same thing in regard to the rapid convalescence in the cases he had seen here.

*Dr. Lockwood* believed the fever mentioned by Dr. Rochester to be a remittent of a low type. Had often been struck by the difference presented by the same disease in different years, and under varying conditions of the atmosphere. As bearing on the point, he related briefly four cases of scarlatina which had occurred in one family under his charge, and which had all recovered, whereas, a few years since, he would have expected to lose at least three of them. One of them had as a sequel to the fever, general anasarca to an unusual extent, being enormously swollen and unable to lie down, and making water. with great difficulty, even in a sitting posture. Measured the urine for forty-eight hours; under the use of iron and the usual treatment for such cases, and only got an ounce in each twenty-four hours. Then commenced giving the rust, which he had exhibited here, and in the first twenty-four hours obtained a pint of urine, and the child convalesced well. Had never seen a more marked case. Also related a marked case of typhoid, which recovered without medication. Believed there was often too much medicine given in such cases.

*Dr. Strong* would say in reference to Dr. Miner's remarks, that he had often seen typhoid fever without diarrhœa, but believed there was generally disease of Peyer's glands, hence he thought we must cling to that as a means of diagnosis; but had often seen it without eruption. Wished chiefly to call attention to the general painlessness of all forms of disease now prevailing. These fevers were for the most part painless. Related a case of pleuro-pneumonia, in which the pneumonia had reached the stage of hepatization, and with effusion of serum into the pleura, which had been perfectly painless.

*Dr. Gay*, as chairman of the committee, appointed at last meeting, on the root presented by Dr. Lockwood, presented the following report:

The committee appointed at the last meeting, to whom was assigned the duty of ascertaining the genus of the plant exhibited by Dr. Lockwood beg leave to report that the standard works on botany, viz: Graey, Tory, and Wood, have been consulted, that we have also consulted with Hon. G. W. Clinton, David F. Day, Esq., and other distinguished botanists of the State, all of whom concur in recognizing the plant in question as the Asclepia incarnata, (swamp milkweed.) Prof. Asa Gray describes two species of the Apocynum, and fourteen species of the Asclepias. Although the two genera present many points of resemblance, they are so essentially different as to be classified under two separate orders.

Some of the distinctive points upon which these two orders are founded, your committte will mention.

The corolla of the Apocynum cannabinum is bell-shaped, the color a greenish white, the stamens arise from the corolla. There are two follicles, one sometimes abortive, long and linear, leaves acute at both ends, not always alternate.

The corolla of the Asclepias incarnata is *reflexed*, of a reddish purple color; the stamens are inserted at the base of the corolla ; there are two follicles, oval, and much larger than the pods of the other genus ; leaves always opposite, acute at the end, somewhat cordate at the base, and longer petioled than the former. The Asclepias is of a much coarser texture than the Apocynum. The first, alone, is sufficient to distinguish the one genus from the other.

The committee would suggest that our works on materia medica are defective in their description of our indigenous plants, and should not be taken as authority.

We have pleasure in according to Dr. Lockwood the credit of having made a valuable contribution to the materia medica by the introduction of this plant to the notice of the profession, since we have no doubt it possesses all the medical properties ascribed to it by that gentleman.

All of which is most respectfully submitted. C. C. F. Gay,
P. H. Strong,
Joseph A. Peters,
*Committee.*

*Dr. Peters* in agreeing to the report wished also to say that the fact that the qualities ascribed by Dr. Lockwood to the root in question were not doubted by the committee, in his opinion made the subject of more value than if they were only ascribed to the A. Cannabinum.

*Dr. Boardman* called attention to the prevalent enlargement and purple redness of the glands of the throat in cases of fever now prevailing. Had used with good effect a gargle containing capsicum,

*Dr. Rochester* reported the prevalence of fever, diarrhœa and dysentery. Dr. Strong the same.

*Dr. Strong* gave notice of an amendment to the by-laws.

Adjourned. JOSEPH A. PETERS, *Secretary.*

